# Development and validation of a risk-prediction nomogram for in-hospital mortality in adults poisoned with drugs and nonpharmaceutical agents: An observational study

**DOI:** 10.1097/MD.0000000000006404

**Published:** 2017-03-24

**Authors:** Catalina Lionte, Victorita Sorodoc, Elisabeta Jaba, Alina Botezat

**Affiliations:** a Internal Medicine and Clinical Toxicology Department, “Grigore T. Popa” University of Medicine and Pharmacy; b Statistics Department, FEAA, “Al. I. Cuza” University; c Romanian Academy—“Gh. Zane” Institute for Economic and Social Research Teodor Codrescu No. 2, Iasi, Romania.

**Keywords:** drugs, mortality, nomogram, nonpharmaceutical agents, poisoning, prediction

## Abstract

Acute poisoning with drugs and nonpharmaceutical agents represents an important challenge in the emergency department (ED).

The objective is to create and validate a risk-prediction nomogram for use in the ED to predict the risk of in-hospital mortality in adults from acute poisoning with drugs and nonpharmaceutical agents.

This was a prospective cohort study involving adults with acute poisoning from drugs and nonpharmaceutical agents admitted to a tertiary referral center for toxicology between January and December 2015 (derivation cohort) and between January and June 2016 (validation cohort). We used a program to generate nomograms based on binary logistic regression predictive models. We included variables that had significant associations with death. Using regression coefficients, we calculated scores for each variable, and estimated the event probability. Model validation was performed using bootstrap to quantify our modeling strategy and using receiver operator characteristic (ROC) analysis. The nomogram was tested on a separate validation cohort using ROC analysis and goodness-of-fit tests.

Data from 315 patients aged 18 to 91 years were analyzed (n = 180 in the derivation cohort; n = 135 in the validation cohort). In the final model, the following variables were significantly associated with mortality: age, laboratory test results (lactate, potassium, MB isoenzyme of creatine kinase), electrocardiogram parameters (QTc interval), and echocardiography findings (E wave velocity deceleration time). Sex was also included to use the same model for men and women. The resulting nomogram showed excellent survival/mortality discrimination (area under the curve [AUC] 0.976, 95% confidence interval [CI] 0.954–0.998, *P* < 0.0001 for the derivation cohort; AUC 0.957, 95% CI 0.892–1, *P* < 0.0001 for the validation cohort).

This nomogram provides more precise, rapid, and simple risk-analysis information for individual patients acutely exposed to drugs and nonpharmaceutical agents, and accurately estimates the probability of in-hospital death, exclusively using the results of objective tests available in the ED.

## Abstract 2

Supplemental Digital Content is available in the text

## 1 Introduction

Acute poisoning is potentially life-threatening and is an important medical emergency. Nomograms are widely used in different clinical settings, to indicate the probability of an event, such as death or disease recurrence, primarily by reducing statistical predictive models to a single numerical estimate tailored to the individual patient profile.^[1]^ For example, in oncology, nomograms can help determine cancer prognosis^[2]^ and offer an accurate individualized prediction of survival,^[3]^ and in neonatology nomograms are used to assess the risk for severe nonphysiologic hyperbilirubinemia after neonates discharge.^[4]^ They reduce statistical predictive models into a single numerical estimate, tailored to the profile of an individual patient, indicating the probability of an event, such as death or recurrence.^[1]^ In clinical toxicology, several nomograms have been developed, including the Done nomogram, indicating the severity of toxicity based on 6-hour levels of non-enteric-coated aspirin,^[5]^ currently with limited clinical use; the Rumack–Matthew nomogram, for antidote therapy decision in acetaminophen overdose^[6]^; the QT nomogram, predictive for arrhythmogenic risk of drug-induced QT prolongation,^[7]^ particularly in antipsychotic,^[8]^ and antidepressant overdoses^[9]^; and graphical nomograms predicting drug concentration.^[10]^ To be relevant for clinical practice, the accuracy of a risk assessment tool should be greater than that of a practitioner's assessment in the emergency department (ED).^[11]^

Our aim was to construct and validate a simple, accurate, and widely applicable nomogram offering an early estimate of the risk of in-hospital mortality, using objective data, immediately available upon presentation to the ED, derived from a population of subjects following acute poisoning with drugs and nonpharmaceutical agents, irrespective of the dose, route of exposure, or mechanism of toxicity. The emergency physician could use this simple tool to identify patients with acute poisoning at risk of death immediately after presentation, and optimize patient management by referring patients to an intensive care unit (ICU), to prevent mortality.

## 2 Materials and methods

### 2.1 Study design

**Figure 1:**
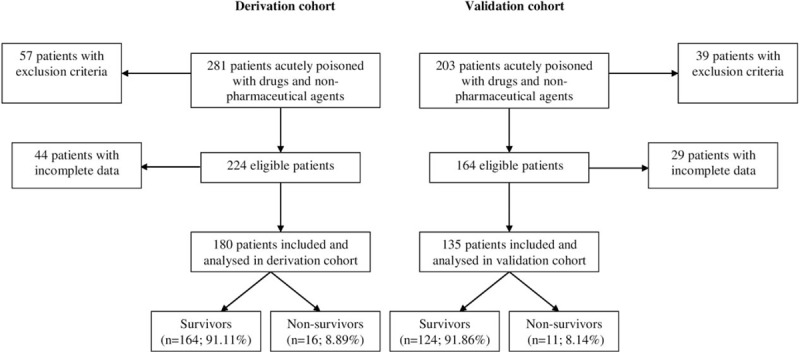
Patient flow diagram.

This paper presents the results of a prospective cohort study involving patients with acute poisoning, conducted in a tertiary referral center for toxicology (Fig. 1). Enrollment occurred between January 2015 and December 2015 (derivation cohort) and between January 2016 and June 2016 (validation cohort). The study was funded by an internal research grant awarded by the “Grigore T. Popa” University of Medicine and Pharmacy Iasi, Romania. This study complied with the principles of the Declaration of Helsinki and was approved by the “Grigore T. Popa” University of Medicine and Pharmacy's Commission for Research Ethics, and by the “Sf. Spiridon” Clinical County Emergency Hospital's Ethics Committee. The study complied with the transparent reporting of an observational cohort study (STROBE) and with a multivariable prediction model for individual prognosis (TRIPOD) statement.^[12]^

### 2.2 Study setting and population

The setting for this study was an urban tertiary center with over 85,000 ED visits annually. Patients eligible for enrollment were over 18 years of age, with acute poisoning as the primary reason for hospital admission, and with admission occurring within 12 hours of exposure to a drug or chemical substance. We enrolled consecutive patients hospitalized in the ICU, or in a non-ICU ward, after obtaining a signed informed consent from the patient or the family (in the case of an unconscious patient). The following were exclusion criteria: lack of a signed informed consent, age under 18 years, diagnosis of diabetes, concomitant acute pathology associated with poisoning (such as trauma and burns, including chemical burns), or incomplete data.

### 2.3 Study protocol

Procedures were identical in the derivation and validation phases of the study. The following data were collected from all patients participating in the study: baseline characteristics, vital signs, mental status, underlying diseases, the type of poison exposure, the intent of the poisoning (self-harm or accident), coingestion of ethanol, laboratory test results, electrocardiogram (ECG) and emergency echocardiography parameters, medical complications, antidote treatment patterns, ICU admission days, and in-hospital outcome. The patients were only followed up during their hospitalization. The derivation cohort for nomogram development consisted of the 180 patients admitted between January 2015 and December 2015, with complete recordings of the parameters set out above.

A standardized data collection form was designed to retrieve all the relevant information on socio-demographic data (age, sex, residence, ethanol exposure, history of chronic disease), baseline laboratory data, and biomarkers. Clinical scores, such as the Glasgow Coma Scale (GCS) and the Poisoning Severity Score (PSS), were recorded for all patients. In a subset of 20 patients (11.1%), blinded, independent second raters duplicated data collection to assess reliability.^[11]^

A blood sample was collected immediately after ED presentation to evaluate arterial blood gases, routine hematology and biochemical analysis, and cardiac biomarkers assessing myocardial injury.^[13]^ The results were obtained using ABL 90 (Radiometer, Denmark), PATHFAST Cardiac Biomarker Analyser (LSI Medience Corporation, Tokyo, Japan), Sysmex XT-4000i—Automated Hematology Analyzer (Sysmex Corporation, Tokyo, Japan), and ARCHITECT c16000 clinical chemistry analyzer (Abbott Laboratories, Abbott Park, Illinois, USA). A 12-lead ECG (to determine ST-T changes, PR, and QTc intervals) was recorded in the ED upon presentation. The QTc was calculated using the Bazett formula, and was considered prolonged if greater than 0.44 seconds.^[7]^ A transthoracic echocardiography using Fukuda Denshi Full Digital Ultrasound System UF-850XTD (Fukuda Denshi Co, Ltd, Tokyo, Japan) was performed in the ED by a cardiologist and an internal medicine specialist with competence in basic echocardiography, to assess systolic and diastolic function of the left ventricle (LV), as well as volume status, according to guideline recommendations.^[14]^ The results were compared, the intra- and inter-reader variability was tested, and no statistical differences between the operators were noted.

### 2.4 Validation cohort

To examine the generalizability of the model, an external validation was performed using a separate cohort of 203 consecutive patients with acute poisoning after exposure to drugs or nonpharmaceutical agents, hospitalized in the same institution between January 2016 and June 2016, and 135 patients with complete data to score all the variables in the established nomogram were analyzed. No data from the validation cohort were used to derive the nomogram, and no data from the derivation cohort were used to validate it.

### 2.5 Key outcome measures

Patient status upon hospital discharge was defined as follows: survivors, defined as patients who survived and were discharged with stable vital signs, with no specific complaints, and with all complications resolved during hospitalization; nonsurvivors, defined as patients who died during hospitalization.

### 2.6 Data analysis

Each variable distribution was presented as mean ± SD, or median with interquartile range, or frequency. The Student *t* test or Mann–Whitney *U* test for numerical variables, as well as the *χ*^*2*^ test and Cochrane statistic for categorical variables, were used to detect significant differences between survivors and nonsurvivors. To evaluate the association between patient data and mortality, we first applied simple binary logistic regression for each variable with significant differences between the 2 groups. Then we applied binary logistic regression on clusters of variables characteristic for systems and organs. We selected significant variables from each cluster, which were included in the final model. Odds ratios (OR) with confidence intervals (CI) were calculated. Goodness-of-fit for multivariate models was confirmed using the Hosmer and Lemeshow test. Based on these results, we generated the nomogram. The receiver operating characteristic (ROC) methodology was used to assess the discriminative power of the nomogram. ROC analyses were expressed as curve plots and calculated area under the curve (AUC) with 95% CI and the associated *P* value representing the likelihood of the null hypothesis (AUC = 0.5). Statistical analyses were performed using SPSS (version 22.0; SPSS Inc, Chicago, IL). We used STATA/SE 13.0, and the nomolog program to generate a Kattan-style nomogram, which is a nomogram for binary logistic regression predictive models.^[15]^ The length of the line corresponding to a given variable correlated positively with the importance of the variable.^[15]^ Internal validation was performed using the bootstrap method. The probability derived from the nomogram for all subjects was verified and compared with the value of the probability estimated using the logistic model.

During external validation of the nomogram, the death risk probability for each patient was calculated using the established nomogram and logistic regression was performed using the predictive variables derived from the validation cohort. These probabilities were subjected to ROC analyses. In addition to comparing the discrimination ability of the AUC, we also calculated the positive predictive value (PPV) and the negative predictive value (NPV) of the scores calculated by the nomogram for the validation cohort. A 2-sided *P* value < 0.05 was deemed significant.

## 3 Results

### 3.1 Patient characteristics and survival

Among the 388 eligible patients (Fig. 1), 180 were included in the derivation cohort, and 135 patients were included in the validation cohort. We excluded 73 patients with incomplete data from the analysis. The patient's demographics, clinical characteristics upon admission, laboratory data, and clinical outcomes are reported for survivors and nonsurvivors in Table 1.

**Table 1:**
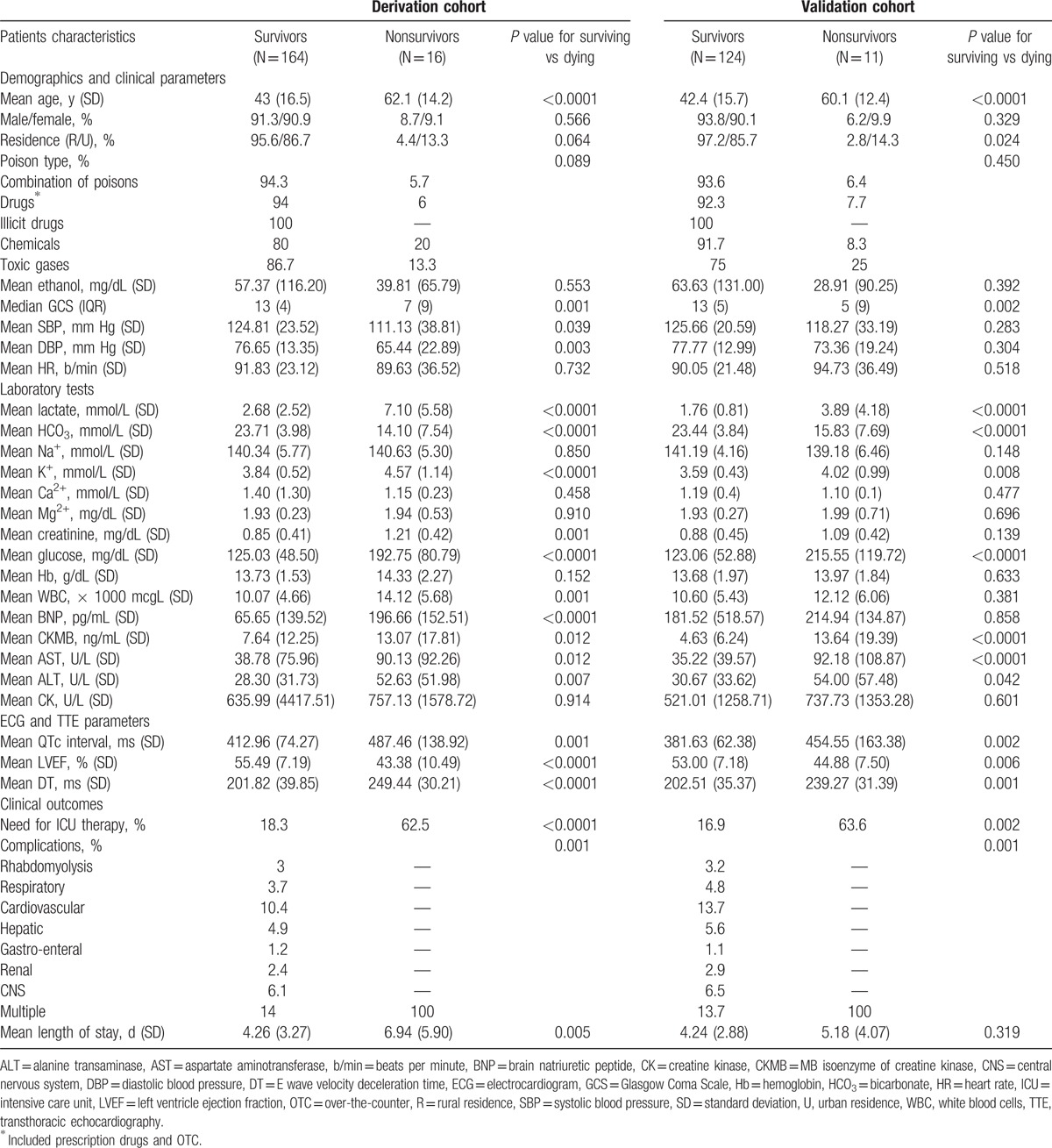
Baseline patient demographics, clinical and laboratory characteristics, and outcomes.

The mean age for both cohorts was 44 years (range, 18–91 years), 50.5% of the subjects were women, all the patients were Caucasian, and 51.42% had rural residence. The time interval between toxin exposure and presentation to the ED ranged from 0.5 to 6.5 hours. The leading cause of poisoning was acute exposure to a combination of poisons (29.4% in the derivation cohort and 34.8% in the validation cohort, respectively). Among the patients in the derivation cohort, the drugs most frequently involved were: sedative hypnotics (13.9%); illicit drugs, including opiates (5%); antidepressants (3.9%); anticonvulsants (3.9%); cardiovascular medication (3.9%); NSAIDs, including salicylates (3.9%); antipsychotics (2.8%); and acetaminophen (2.8%) (see supplemental content). The distribution of nonpharmaceutical poisons was as follows: pesticides and herbicides (11.7%); carbon monoxide (8.3%); toxic alcohols, other than ethanol (5%); other chemicals, such as formaldehyde or hydrocarbon mixtures (2.2%); and rat poison (1.2%). The majority of the cases were due to self-poisoning, with only 23 cases (7.3%) being accidental poison exposures.

The overall in-hospital mortality rate was 8.57% (n = 27), providing an adequate number of events to evaluate predictors (see supplemental content). The direct cause of death was multiple complications involving at least 2 vital organs (dysrhythmias, toxic-induced myocardial injury, refractory shock, acute respiratory distress syndrome, and multiple organ failure). In both cohorts, deaths were recorded in patients with acute poisoning involving chemicals (10 patients; 3.2%), drugs (7 patients; 2.2%), a combination of poisons (6 patients; 1.9%), and toxic gases (4 patients; 1.3%).

### 3.2 Univariable and multivariable analysis

Out of the 180 patients of the derivation cohort, there were 16 nonsurvivors (8.89%). Univariable predictors of in-hospital mortality are shown in Table 2. On multivariable analysis, only 6 of the 20 candidate variables remained predictive of mortality (Table 3). The following variables independently correlated with mortality: age, lactate upon ED presentation, potassium (K^+^), initial MB isoenzyme of creatine kinase (CKMB) upon ED arrival, the QTc interval on initial ECG, and the E wave velocity deceleration time (DT) on the echocardiography performed in the ED. Although other variables (including, urban residence, glucose level, and left ventricular ejection fraction) were predictive for mortality on univariable analysis, these were not included in the final model because the association was not statistically significant. Blinded duplicate assessments were performed in 20 patients (researcher only: 7 patients; physician only: 10 patients; both physician and researcher: 3 patients), and there were no statistically significant differences in the interexaminer assessments.

**Table 2:**
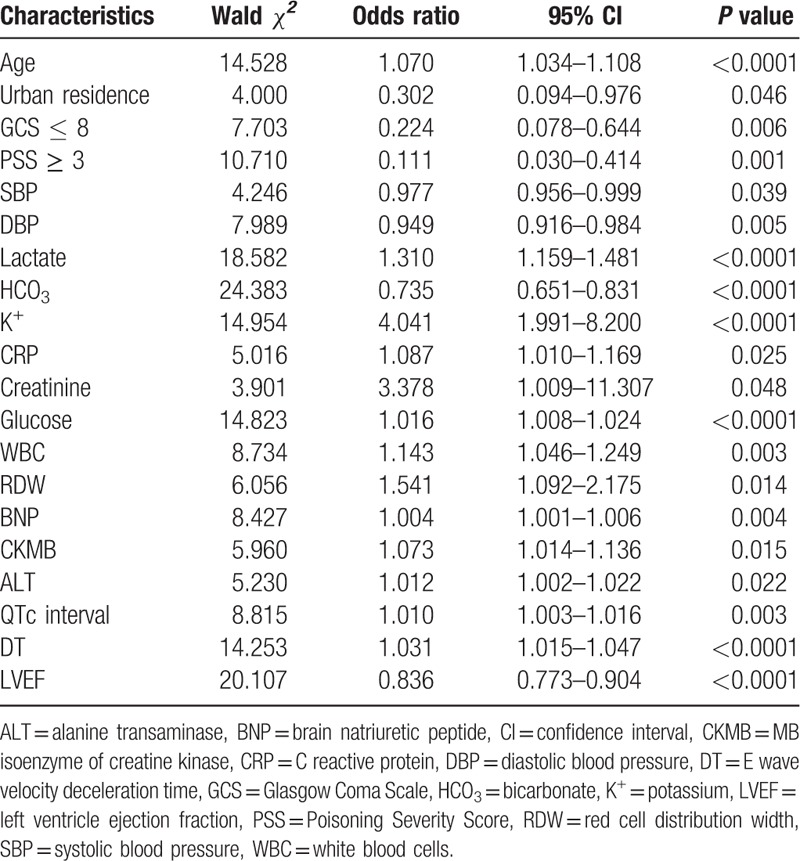
Selected factors using univariate analysis for building the model.

**Table 3:**
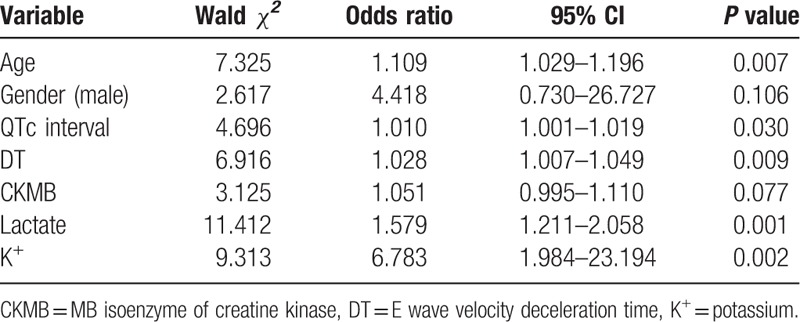
In-hospital mortality model.

### 3.3 Nomogram development

Binary logistic regression analysis indicated that death probability in acute poisoning can be estimated using the following 6 significant predictor variables: age, initial lactate, K^+^, initial CKMB, the QTc interval, and DT. We added sex as a further demographic characteristic (Table 3), to use the same nomogram for male and female patients. ROC curves had validated discriminatory power of predictive variables for mortality. The areas under the curves were: DT—0.84 (95% CI 0.74–0.94, *P* < 0.001); age—0.80 (95% CI 0.70–0.91, *P* < 0.001); initial lactate—0.74 (95% CI 0.58–0.90, *P* = 0.002); initial CKMB—0.69 (95% CI 0.57–0.81, *P* = 0.012); QTc interval—0.68 (95% CI 0.53–0.83, *P* = 0.018); K^+^—0.66 (95% CI 0.47–0.84, *P* = 0.038).

This analysis indicated that all predictor variables had good discriminatory power. The following variables had a high value of AUC, predicting mortality with excellent discrimination (AUC > 0.80): DT (cutoff point 232 msec; sensitivity of 87.5% and a specificity of 79.9%) and age (cutoff point 53 years; sensitivity of 87.5% and a specificity of 72%).

**Figure 2:**
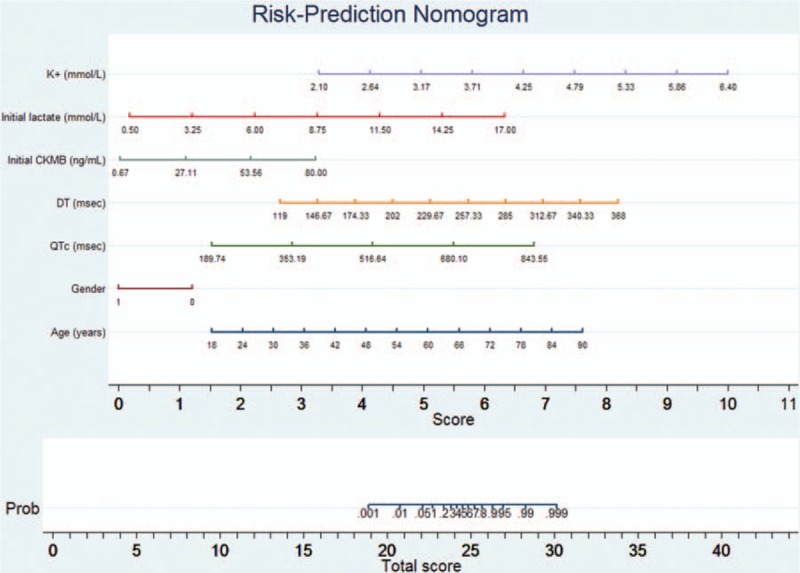
Risk-prediction nomogram for mortality in acute poisoning with drugs and nonpharmaceutical agents incorporating age (y), sex (1 male, 0 female), QTc interval (msec), DT (msec), initial CKMB (ng/mL), initial lactate levels (mmol/L), and K^+^ levels (mmol/L). CKMB = MB isoenzyme of creatine kinase, DT = the E wave velocity deceleration time, QTc = corrected QT interval.

Using the 6 independent risk factors, in addition to sex, we developed a nomogram that predicts in-hospital mortality (Table 4). The nomogram was characterized by 1 scale corresponding to each variable, a score scale, a total score scale, and a probability scale (Fig. 2).

**Table 4:**
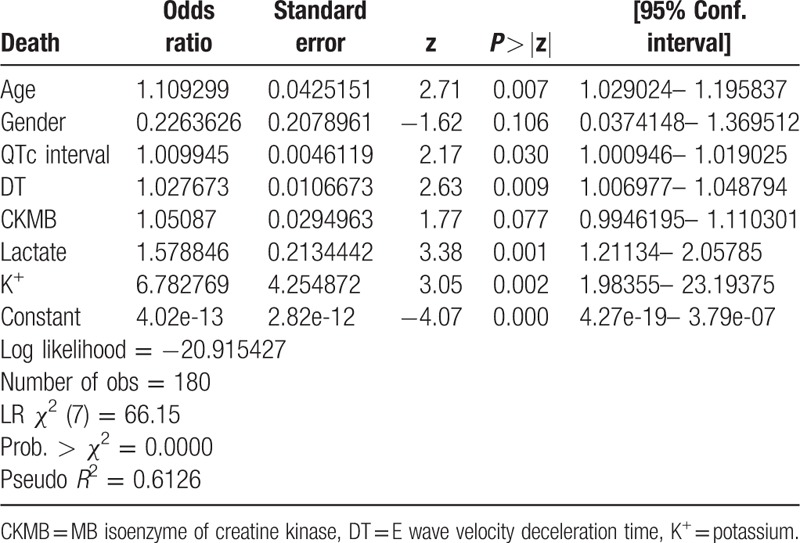
The risk-prediction nomogram.

The use of the nomogram is simple, and involves 3 steps. First, on the scale for each variable, the value corresponding to a specific patient is read and the score scale is used to calculate the scores for all variable values. Second, the total score is calculated by adding up all the scores obtained in the previous step, and its value is identified on the total score scale. Finally, the probability of an event corresponding to the total score of the subject is read on the probability scale.

**Figure 3:**
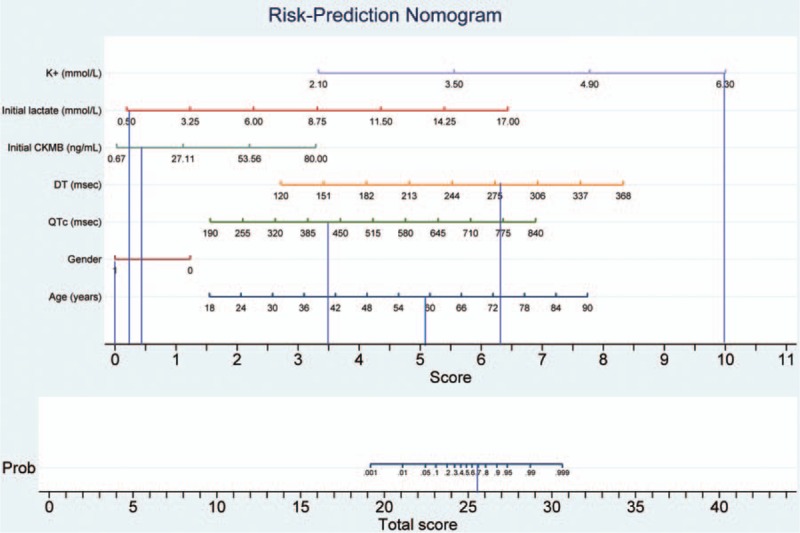
Risk-prediction nomogram in a patient with acute poisoning from a toxic alcohol that did not survive. A line is drawn downward from the value of each category to the score line. The points are then added to determine the total score, and a line is drawn upward to find the risk of mortality. Death probability estimation: K^+^ (mmol/L): 6.3 − score = 10; initial lactate (mmol/L): 0.9 − score = 0.2; initial CKMB (ng/mL): 7.94 − score = 0.4; DT (msec): 278 – score = 6.3; QTc (msec): 427.36 – score =3.5; sex: 1 (male) – score = 0; age (y): 59 – score =5.1. Total score = 25.5, with a death probability of 0.68. CKMB = MB isoenzyme of creatine kinase, DT = the E wave velocity deceleration time, QTc = corrected QT interval.

**Figure 4:**
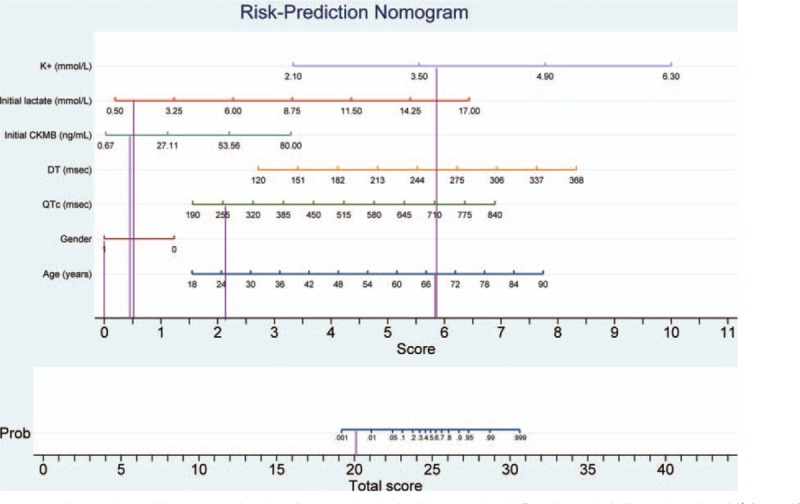
Risk-prediction nomogram in a patient with acute poisoning from toxic alcohol that survived. Death probability estimation: K^+^ (mmol/L): 3.7 − score=5.8; initial lactate (mmol/L): 1.3 − score=0.5; initial CKMB (ng/mL): 7.3 − score=0.4; DT (msec): 251 – score=5.6; QTc (msec): 258.7 – score= 2.1; sex: 1 (male) – score=0; age (y): 68 – score=5.8. The total score = 20.2, with a death probability of 0.004.

For example, 2 unconscious patients were admitted to the ED after acute exposure to an unknown quantity of toxic alcohol. There were no available data regarding the time from poison exposure to ED presentation, and the serum levels of toxic alcohol could not be assessed in the ED. Clinical management was comparable in these cases: both patients were admitted to the ICU and received antidote therapy with ethanol (the only available antidote for toxic alcohol poisoning at that time), underwent hemodialysis for toxin removal, and received supportive therapy. However, the outcomes were different. The first patient died after 9 days in the ICU, the second patient was transferred to the medical ward after 1 day, and discharged home 2 days later. The application of the nomogram to the first patient (Fig. 3) showed a total score of 25.5, with a death probability of 0.68. The death probability calculated using the nomogram was identical to the one estimated by using logistic regression model. The first patient did not survive. The same methodology was applied to the second patient showing a significantly lower death probability; the second patient survived (Fig. 4).

The nomogram was evaluated as a diagnostic test calculating sensitivity, specificity, and positive and negative likelihood ratios. The final model was internally validated using bootstrap resampling.^[16]^

Receiver-operating characteristic analysis indicated that the accuracy of the predicted probability for the model was 97.6% compared with 84% or less, when using a single variable (Table 5).

**Table 5:**
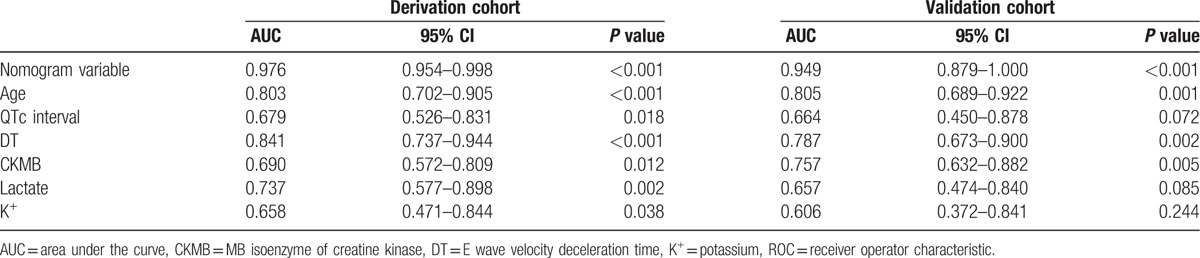
The AUC of the ROC curves for the nomogram and variables from logistic regression model in derivation and validation cohort.

### 3.4 Nomogram use in the stratification of patient risk

**Figure 5:**
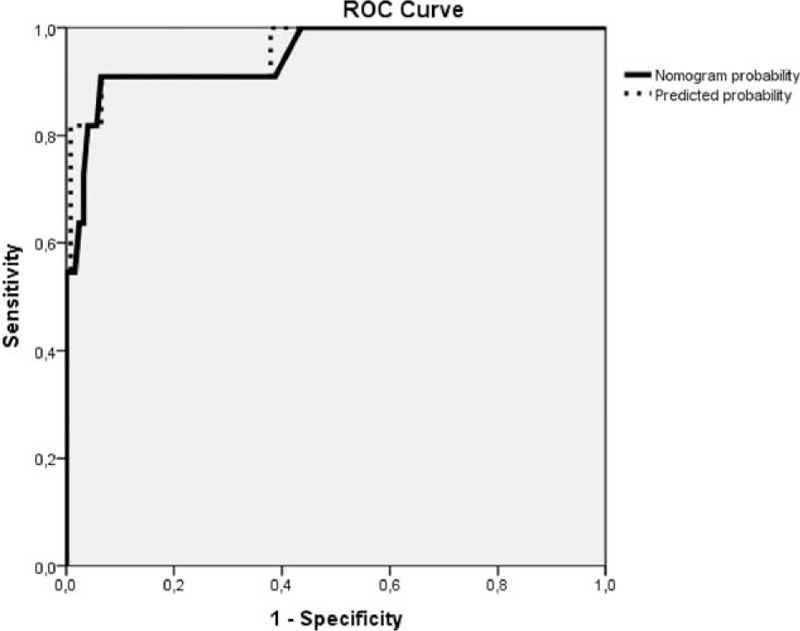
Receiver operating characteristic curves validate the discriminatory power of the nomogram predictive for mortality in the validation cohort. Areas under the curves: nomogram probability – 0.949 (95% confidence interval 0.879–1.000, *P* < 0.001); predicted probability using the developed model–0.957 (95% confidence interval 0.892–1.000, *P* < 0.001).

There were 135 patients and 11 deaths in the validation cohort (8.14% mortality rate). The nomogram was used to assess the risk of mortality for all patients in the validation cohort; the probability indicated by the nomogram was then compared with the probability using the model developed. The AUC for the nomogram was 0.95 (95% CI, 0.88–1, *P* < 0.0001), and the AUC for the model was 0.96 (95% CI, 0.89–1, *P* < 0.0001; Fig. 5), which proved that our logistic regression model and nomogram had superior capability in predicting mortality. For high-risk patients (total score > 24 points), the sensitivity was 90.9%, the specificity was 93.5%, the PPV 100%, and the NPV 96%. For low-risk patients (total score ≤ 24 points), the sensitivity was 81.8%, the specificity was 96%, the PPV 96%, and the NPV 100%.

## 4 Discussion

To our knowledge, our model is the first risk-prediction nomogram attempting to evaluate in-hospital mortality in patients with acute poisoning. In the field of toxicology, the nomograms currently available are used for the identification of the benefits of antidote therapy in acetaminophen-poisoning and for toxicity or arrhythmia risk assessment, with some nomograms also used in clinical practice.^[5–7]^ There have been recent attempts to generate a nomogram for choosing the appropriate duration of hemodialysis in acute methanol poisoning.^[17]^

A nomogram is a graphical representation of a mathematical formula or algorithm incorporating several predictors modeled as continuous variables to predict an end-point, based on traditional statistical methods, such as multivariable logistic regression and Cox proportional hazards analysis.^[1]^ Nomograms also provide superior individualized disease-related risk estimations that facilitate patient management-related decisions. Nomograms are currently the most accurate available tools, with the greatest discriminating characteristics for predicting outcomes in patients with different oncologic pathologies,^[1,3,18]^ or in patients with heart failure.^[19]^

In the present study, we developed a predictive model incorporating demographic, ECG and echocardiography parameters, as well as laboratory data, from a cohort of patients with acute poisoning with different toxins, to determine the mortality risk on admission to the ED.

The significant independent covariates for the mortality risk in the present study were age, DT as a measure of impaired LV diastolic function, initial lactate level, CKMB as a marker of myocardial injury, the QTc interval, and the potassium level. Furthermore, we used sex as a categorical variable.

Using these factors, we constructed a nomogram providing more precise, simple, and rapidly available risk-analysis information for individual patients acutely exposed to a poison, irrespective of type (drug, chemical, or gas), toxicokinetics, dosage, and route of entry. The information is based on objective markers, such as laboratory tests and imaging parameters, and not on clinical scales which may be applied subjectively by physicians according to different levels of expertise. In cases of acute poisoning involving unresponsive patients in the ED, with no knowledge of the type, dose, or serum levels of the offending toxin, this predictive nomogram may aid emergency physicians to identify high-risk patients more promptly, enabling the administration of specific or aggressive therapy, or the immediate referral of the patient to an ICU with advanced capacity, to reduce mortality. Nomogram use may not only facilitate early management decision making, but may also minimize unnecessary tests and expenses. In the example presented in the Results section, the use of the nomogram in the ED would have identified, on admission, the patient with a low risk of death, and would have helped to choose a different approach, for example, admission to a non-ICU ward and avoidance of hemodialysis.

The in-hospital mortality predictors detected in our model are consistent with other published reports of patients hospitalized with acute poisoning. Retrospective studies found that prolonged QTc interval, older age, increased arterial lactate upon admission, or myocardial injury were associated with in-hospital mortality following exposure to different types of poison.^[20–24]^ Our previous research showed that there are objective indicators, available in the ED, that can predict a poor outcome in patients exposed to systemic poisons, such as cardiac biomarkers, and lactate.^[25]^

In this prospective study, we confirmed the predictive role for death of increased age, initial lactate level, CKMB, and prolonged QTc interval; we also identified new variables, such as DT, and K^+^ levels, which accurately predicted the mortality risk. These findings confirm the relevance of these variables as prognostic factors in a representative sample of the population with acute poisoning due to a range of toxins.

Acute poisonings represent a problem in both developed and developing regions worldwide. In Romania, epidemiological data suggest that acute drug poisoning in suicide attempts is the most common reason for hospitalization of patients with poisoning (97.27%) and poisoning more frequently occurs due to a combination of drugs (32.92%), with a mortality rate of 0.3%.^[26]^ Self-poisoning with organophosphate pesticides in our area showed a mortality rate of 3.8%.^[27]^ Most of the patients in our study with acute poisoning had attempted suicide, using drugs or a combination of poisons, comparable with the distribution reported in studies from the United States, or Central Europe.^[28,29]^

High troponin levels have been reported to be associated with an increased mortality risk in acute drug poisoning,^[30]^ although this finding could not be corroborated by our model. However, the number of patients with readings above the normal range may not have been sufficiently large to detect any evidence of this association after acute exposure to heterogeneous drugs and nonpharmaceutical agents.

Our results confirmed that the parameters assessing early acute myocardial injury, such as prolonged DT, which reflects diastolic dysfunction, are predictive of mortality. This is consistent with the findings reported for carbon monoxide poisoning, where diastolic dysfunction precedes systolic dysfunction of the left ventricle, even in the absence of ECG changes.^[31,32]^

We also found that potassium levels have a potential role in mortality risk-prediction in patients with acute poisoning, contrary to previous research on self-poisoning with different types of medication which failed to demonstrate that abnormal K^+^ levels were a life-threatening event requiring emergency treatment and/or ICU admission.^[33]^ This finding may be explained by the heterogeneity of the poisons used and the proportion of nonpharmaceutical agents (33.3%) in our cohort. However, researchers have demonstrated that an increased in-hospital mortality rate is significantly associated with severe underlying disease and coexisting medical conditions, as well as with a severe increase in K^+^ levels.^[34]^

Although hyperglycemia is not a common feature of overdose,^[35]^ the admission levels of blood glucose following acute poisoning may be associated with clinical outcome.^[36]^ The association between glucose levels and mortality in patients with organophosphate and methanol poisoning has been proven.^[37,38]^ We did not include glucose levels in the nomogram, based on the results of multivariate analysis.

The mortality rate in our cohort was comparable to that on reported in other prospective observational studies (9.7%) poisoning due to a combination of drugs,^[39]^ and lower than pesticide-related fatalities (25.31%) reported in retrospective studies.^[40]^ However, our mortality rate was higher than that reported in 2012 by the American Association of Poison Control Centers, concluding that only 1% of fatalities were exposure-related.^[41]^ A possible explanation for the higher fatality rate in our cohort could be the fact that some antidotes are unavailable in our country, such as 4-methylpyrazole (for toxic alcohols), and Digoxin Immune Fab (for digitalis glycosides).

Our results demonstrated the benefits of the nomogram used as a decision making-support tool by emergency physicians in patients with acute poisoning with drugs and nonpharmaceutical agents. This risk-prediction nomogram may have an advantage over traditional tools, such as GCS, PSS, or other clinical scores, because the association between predictors (age, sex, QTc interval, DT, CKMB, lactate and K^+^) and the predicted variable (death) is visible at a glance. This advantage may be particularly useful in areas where the nomogram user can choose the values of the covariates (e.g., a physician making management decisions involving several factors).

Some limitations of this study should be mentioned. First, the data were collected from a single tertiary center. Findings from our study may not be generalizable to other populations of patients with acute poisoning, although the epidemiological data in our area are consistent with those reported in different regions of the world.^[28,29]^ Second, this was an observational study, so it is possible that there are unmeasured systematic biases that are specific to the region. However, there is no a priori reason to assume that local practices or facilities differ substantially from elsewhere, and demographic effects are incorporated in the nomogram itself. It is reasonable to assume that the nomogram can be broadly applicable, at least within a nondiabetic adult population with acute poisoning with drugs and nonpharmaceutical agents. Third, further nomograms, as well as improvements in existing nomograms, are required, as none of the existing nomograms are able to make predictions with perfect accuracy. We could not account for the impact of the time to ED presentation, after acute exposure, the toxicokinetics, and the serum poison level on mortality in our cohort. We did not use a simplified model without including DT, despite the fact that performing bedside echocardiography on admission may be difficult in some EDs. Finally, no data on outcome after hospital discharge were available, and death occurring after discharge may have been missed. Novel biomarkers, larger data sets, improved data collection methods, and more sophisticated modeling procedures are needed to improve predictive accuracy.

We intend to continue the research to validate this nomogram in a separate prospective trial, involving a larger dataset of patients with acute poisoning and including poison with local effects, as well as diabetic subjects.

## 5 Conclusions

We developed a 7-variable risk-prediction nomogram based on demographic, routine laboratory tests, and ECG and echocardiography parameters, which accurately predicts the probability of in-hospital mortality for nondiabetic subjects acutely exposed to drugs and nonpharmaceutical agents, exclusively from the objective tests available in the ED. This nomogram used in cases of acute poisoning with drugs and non-pharmaceutical agents has the potential to identify high-risk patients upon presentation to the ED. Further research is required to demonstrate how this nomogram applies to other populations (for example, to subjects under 18 years, different ethnic groups, and patients with caustic exposure) and to elucidate how the incorporation of this tool into clinical practice improves the care or use of resources in patients with acute poisoning.

## Supplementary Material

Supplemental Digital Content
