# Supplementary material for: Development and validation of a risk-prediction nomogram for in-hospital mortality in adults poisoned with drugs and nonpharmaceutical agents: An observational study
Source: Medicine (Baltimore). 2017 Mar 24;96(12):e6404. doi: 10.1097/MD.0000000000006404 (PMC5371475; doi:10.1097/MD.0000000000006404)
Supplement: Supplemental Digital Content [file medi-96-e6404-s001.docx]

**Table online 1.** Xenobiotics involved in the acute poisoning.

| Derivation cohort  (180 patients) | Poison involved | N | Percent | Validation cohort  (135 patients) | N | Percent |
| --- | --- | --- | --- | --- | --- | --- |
|  | Ethanol co-ingestion | 74 | 41.1 |  | 48 | 35.6 |
|  | Combinations (multiple drugs; drug-toxin) | 53 | 29.4 |  | 47 | 34.8 |
|  | Single agent exposure | | |  | Single agent exposure | |
|  | Acetaminophen | 5 | 2.8 |  | 1 | .7 |
|  | Salicylates | 4 | 2.2 |  | 1 | .7 |
|  | NSAID's | 3 | 1.7 |  | - | - |
|  | COX-2 inhibitors | 1 | .6 |  | - | - |
|  | Antidementia drugs | 2 | 1.1 |  | 1 | .7 |
|  | Benzodiazepines | 13 | 7.2 |  | 8 | 6 |
|  | Barbiturates | 5 | 2.8 |  | 1 | .7 |
|  | Miscellaneous anxiolytics, sedatives and hypnotics | 7 | 3.9 |  | 2 | 1.5 |
|  | Antipsychotics | 5 | 2.8 |  | 3 | 2.2 |
|  | Antidepressants | 7 | 3.9 |  | 9 | 6.7 |
|  | Cardiovascular drugs (CCBs, BBs, digitalis glycosides, ACEI) | 7 | 3.9 |  | 8 | 6 |
|  | Antiepileptics | 7 | 3.9 |  | 3 | 2.2 |
|  | Drugs of abuse (cannabis, ethnobotanicals, ecstasy, heroin) | 7 | 3.9 |  | 5 | 3.7 |
|  | Narcotic analgesics | 2 | 1.1 |  | 2 | 1.5 |
|  | Cocaine | 1 | .6 |  | - | - |
|  | Ethylene glycol | 4 | 2.2 |  | 9 | 6.7 |
|  | Methanol | 5 | 2.8 |  | 8 | 6 |
|  | Isoniazid | 1 | .6 |  | - | - |
|  | Organophosphate compounds | 11 | 6.1 |  | 10 | 7.4 |
|  | Organochlorine compounds | 6 | 3.3 |  | 5 | 3.7 |
|  | Miscellaneous pesticides & herbicides | 4 | 2.2 |  | 2 | 1.5 |
|  | Rat poison | 1 | .6 |  | - | - |
|  | Hydrocarbon mixtures | 2 | 1.1 |  | 1 | 0.7 |
|  | Formaldehyde | 2 | 1.1 |  | 1 | 0.7 |
|  | Toxic gases and fumes | 15 | 8.3 |  | 8 | 6 |

NSAID's, non-steroidal anti-inflammatory drugs; COX-2, cyclooxygenase-2; CCBs, calcium channel blockers; BBs, beta-blockers; ACEI, angiotensin-converting enzyme inhibitor.

Treatment received in the emergency room

Thirty-seven (20.6%) patients in the derivation cohort received a specific antidote therapy, 46 (25.6%) patients were treated by activated charcoal, and 2 (1.1%) had a gastric lavage. Ninety-five (52.8%) patients didn’t receive an antidote therapy (either non-existing antidote or unavailable specific antidote in our country).

Among the 16 non-survivors in the derivation cohort, 6 patients received an antidote therapy.

Out of the 11 non-survivors in the validation cohort, 4 patients received an antidote therapy.

**Non-survivor patients:**

*Derivation cohort:*

*-* 4 patients were poisoned with toxic alcohols (2 methanol, 2 ethylene glycol), 3 patients were poisoned with multiple xenobiotics (antidepressants with benzodiazepines, multiple cardiovascular drugs, and pesticides with antipsychotics), two deaths were recorded after lamotrigine poisoning, 2 patients died after carbon monoxide exposure, and two after formaldehyde exposure, followed by acute poisoning with antidepressants, organophosphate pesticides and sedative-hypnotic, each with a case.

*Validation cohort:*

*-* 3 patients were intoxicated with combination of poisons (antidepressants, cardiovascular drugs, and pesticides with formaldehyde), 2 patients were exposed to a toxic alcohol (ethylene glycol), two patients died after acute carbon monoxide exposure, three deaths were recorded after prescription medication poisoning (one with antidepressants, one with lamotrigine, and one with calcium-channel blockers) and one after organophosphate pesticide exposure.
